# Novel mouse monoclonal antibodies specifically recognize *Aspergillus fumigatus* galactomannan

**DOI:** 10.1371/journal.pone.0193938

**Published:** 2018-03-08

**Authors:** Andrey L. Matveev, Vadim B. Krylov, Ljudmila A. Emelyanova, Arsenii S. Solovev, Yana A. Khlusevich, Ivan K. Baykov, Thierry Fontaine, Jean-Paul Latgé, Nina V. Tikunova, Nikolay E. Nifantiev

**Affiliations:** 1 Institute of Chemical Biology and Fundamental Medicine, Siberian Branch of Russian Academy of Sciences, prosp. Lavrentieva 8, Novosibirsk, Russia; 2 N.D. Zelinsky Institute of Organic Chemistry, Russian Academy of Sciences, Leninsky Prospect 47, Moscow, Russia; 3 Novosibirsk State University, Pirogova str., Novosibirsk, Russia; 4 Unité des Aspergillus, Institut Pasteur, Paris, France; Dartmouth College, Geisel School of Medicine, UNITED STATES

## Abstract

A panel of specific monoclonal antibodies (mAbs) against synthetic pentasaccharide β-D-Gal*f*-(1→5)-[β-D-Gal*f*-(1→5)]_3_-α-D-Man*p*, structurally related to *Aspergillus fumigatus* galactomannan, was generated using mice immunized with synthetic pentasaccharide-BSA conjugate and by hybridoma technology. Two selected mAbs, 7B8 and 8G4, could bind with the initial pentasaccharide with affinity constants of approximately 5.3 nM and 6.4 nM, respectively, based on surface plasmon resonance-based biosensor assay. The glycoarray, built from a series of synthetic oligosaccharide derivatives representing different galactomannan fragments, demonstrated that mAb 8G4 could effectively recognize the parental pentasaccharide while mAb 7B8 recognizes its constituting trisaccharide parts. Immunofluorescence studies showed that both 7B8 and 8G4 could stain *A*. *fumigatus* cells in culture efficiently, but not the mutant strain lacking galactomannan. In addition, confocal microscopy demonstrated that *Candida albicans*, *Bifidobacterium longum*, *Lactobacillus plantarum*, and numerous gram-positive and gram-negative bacteria were not labeled by mAbs 7B8 and 8G4. The generated mAbs can be considered promising for the development of a new specific enzyme-linked assay for detection of *A*. *fumigatus*, which is highly demanded for medical and environmental controls.

## Introduction

**Fig 1:**
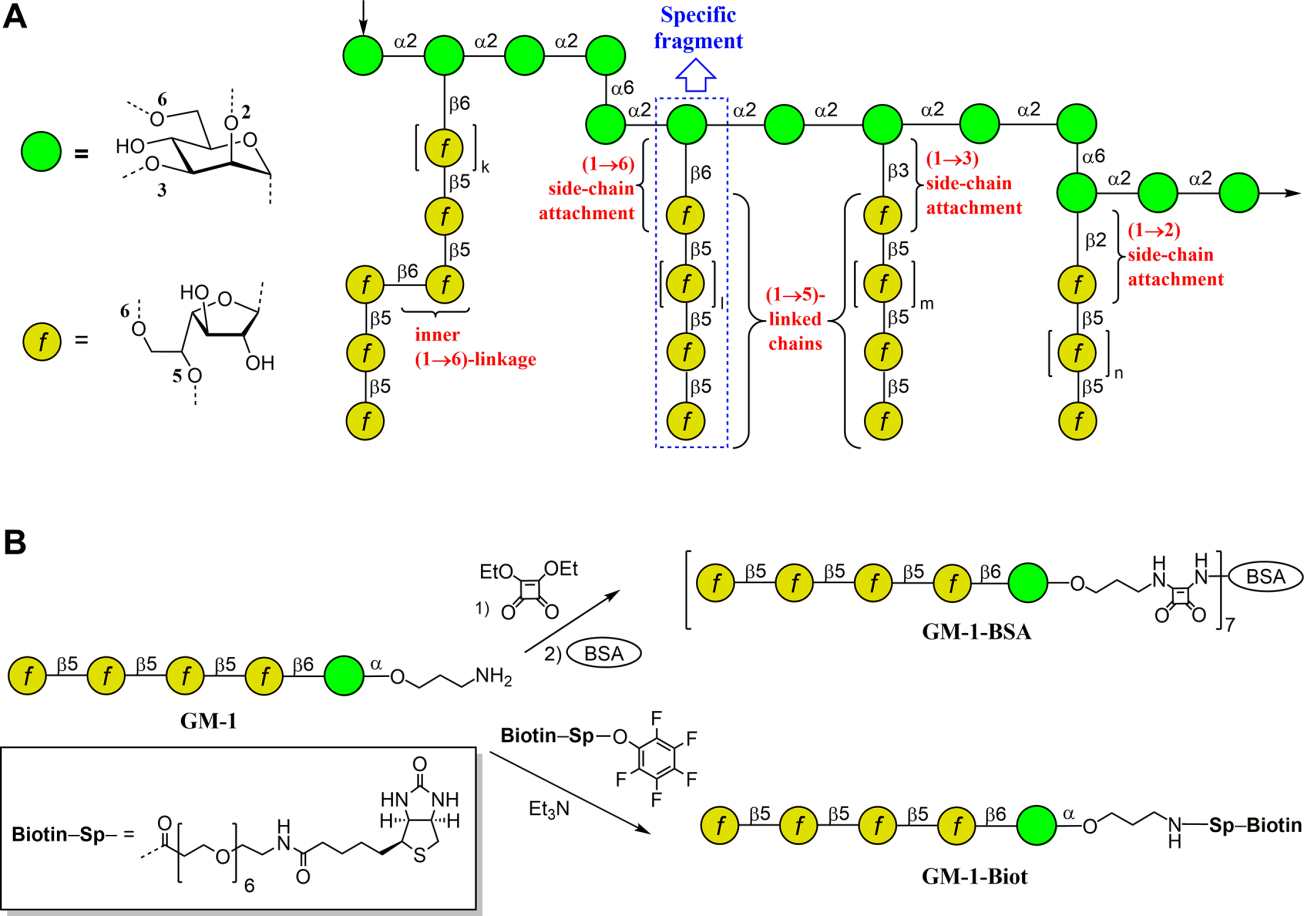
Structure of *Aspergillus fumigatus* galactomannan and its synthetic analogs. (A) Structural fragments of *A*. *fumigatus* galactomannan (summarized from refs. [6] and [8]). (B) Pentasaccharide **GM-1** and its BSA **(GM-1-BSA)** and biotinylated **(GM-1-Biot)** conjugates used in mice immunization and mAb screening. The carbohydrate sequences are represented according to symbol carbohydrate nomenclature [26].

*Aspergillus fumigatus* is the causative agent of a wide range of infections, the most common being allergic bronchopulmonary aspergillosis, local (non-invasive) aspergillosis, chronic pulmonary aspergillosis, as well as invasive aspergilloses [1]. In recent years, invasive pulmonary aspergillosis has been a leading cause of infection-related deaths among immunocompromised patients [2]. This infection often accompanies pulmonary tuberculosis, lung cancer, and chronic bronchitis, and may develop in transplant recipients [2–5]. The major antigenic component secreted by *A*. *fumigatus* into the growth medium is galactomannan–a soluble polysaccharide with molecular weight of approximately 20 kDa [6]. This polysaccharide is also present in glycoproteins as N- and O-glycan moieties and a GPI-anchored lipophosphogalactomannan [7]. Structurally, galactomannan contains a linear mannan core comprising mannotetraose repeating units connected via α-(1→2)- and partly by α-(1→6)- linkages. Some of the α-(1→2)-linked mannoside residues of a mannan backbone have side chains composed of an average of 4 to 5 β-(1→5)-galactofuranoside units attached via β-(1→6)- or β-(1→3)- linkages [6]. Recently, two new structural elements of *A*. *fumigatus* galactomannan have been revealed [8]. These are oligogalactofuranoside side chains containing not only β-(1→5)-linkages, but also one internal β-(1→6)-bond between galactofuranoside residues as well as oligogalactofuranoside side-chains α-(1→2)-attached to the mannan backbone (structural fragments of *A*. *fumigatus* galactomannan are summarized in Fig 1A).

The *A*. *fumigatus* antigens in biological fluids can be detected by a commercial sandwich enzyme-linked immunosorbent assay (ELISA) Platelia *Aspergillus* (Bio-Rad, Marnes-laCoquette, France) [9,10]. This diagnostic tool is widely used; however, the rate of false-positive results for this assay is rather high, and can vary from 5% in adults to 83% in premature infants [11,12]. Other issues that interfer with the detection of galactomannan and reduce the specificity [9,13–15] of this diagnostic tool are antibiotic therapy [16] and dietary factors [17]. These false-positive results are associated with cross-reactive binding of EB-A2 monoclonal antibodies (mAbs) employed in the commercial assay with different non-*Aspergillus* fungi [18–23]. In addition, cross-reactivity with different bacteria, especially with *Bifidobacterium* spp., and members of the normal gastrointestinal microbiota of adults and infants has also been reported [24,25]. This cross-reactivity made it challenging to develop new mAb with improved specificity to be applicable in ELISA for galactomannan detection. The present study reports two new galactomannan-recognizing mAbs developed using BSA-bound (**GM-1-BSA**) and biotinylated **(GM-1-Biot)** conjugates of synthetic pentasaccharide β-D-Gal*f*-(1→5)-[β-D-Gal*f*-(1→5)]_3_-α-D-Man*p* derivative (**GM-1**) (Fig 1B).

## Materials and methods

### Conjugates of synthetic oligosaccharides

The synthesis of oligosaccharides related to galactomannan fragments has been described previously [27,28]. Bovine serum albumin (BSA) conjugate **GM-1-BSA** (Fig 1B) was prepared using the squarate protocol [29]. Thus, diethyl squarate (4 μL, 0.027 mmol) was added to pentasaccharide **GM-1** solution (10.0 mg, 0.011 mmol) in 50% aqueous ethanol (1 mL). The resulting mixture was incubated for 16 h at room temperature. Then triethylamine (3 μL) was added; after 5 h, the solvents were removed. The residue was dissolved in 2 mL water and loaded onto a Sep-Pak C-18 cartridge and washed with water (10 mL). Then, the product was eluted with a gradient of methanol (5% → 20%) in water. The eluate was concentrated, and the residue was lyophilized to give a squarate intermediate (9.5 mg). A solution of this product (4.6 mg, 4.6 μmol) and BSA (15.4 mg) in 3 mL of the buffer solution (250 mL water, 8.8 g KHCO_3_, 6.7 g Na_2_B_4_O_7_·10H_2_O, pH 9) was incubated for 3 days at room temperature. The conjugate was isolated by gel chromatography on a Sephadex G-15 column in water and lyophilized to give 10 mg (50%) BSA conjugate **GM-1-BSA**. MALDI TOF mass spectrum analysis was used to identify the average presence of 8 pentasaccharide ligands per BSA molecule.

To prepare the biotinylated conjugate **GM-1-Biot**, the solution of the parent spacered pentasaccharide **GM-1** (0.116 mmol) in Et_3_N (10 μL) was treated with the active ester of biotin (0.139 mmol) in DMF (0.7 mL) following the biotinylation protocol described previously [30] (as shown in Fig 1B). The mixture was incubated for 18 h at room temperature and then concentrated in vacuum, followed by gel-permeation chromatography on TSK HW-40(S) column (2 × 80 cm) in 0.1 M AcOH that formed the product **GM-1-Biot** with 75% yield. Biotinylated glycoconjugates **1–10**, **GM-2**, **GM-3** (see section «Epitope specificity of selected mAbs» below) used in the creation of glycoarrays on streptavidin-coated plates, were prepared similarly with 65–75% yields starting from the corresponding aminopropyl glycosides [27,28,31], synthesized using pyranoside-*into*-furanoside rearrangement [32,33].

### Animals

Female BALB/c mice were purchased from the animal care facility in the Federal State Research Center of Virology and Biotechnology “Vector”, (Koltsovo, Russia). Mice were housed in plastic cages, 800 cm^2^, (6–10 animals per cage) under normal light-dark cycle. Water and food were provided *ad libitum*. All animal procedures were carried out in accordance with the recommendations for the protection of animals used for scientific purposes (EU Directive 2010/63/EU). The animals were euthanized with overdose of isoflurane (5%). Exposure of isoflurane was continued during one minute after breathing stop. All experiments with animals were approved by the local Bioethics Committee of Institute of Chemical Biology and Fundamental Medicine, Siberian Branch of Russian Academy of Sciences, Novosibirsk, Russia.

### Eukaryotic, fungal, and bacterial cells

SP2/0 myeloma cell line obtained from the EMTC collection (Institute of Chemical Biology and Fundamental Medicine, Novosibirsk, Russia) was cultured in Iscove’s modified Dulbecco’s medium (Invitrogen, USA) supplemented with 10% fetal bovine serum (Biolot, Russia) and antibiotics (0.1 mg/mL streptomycin and 100 IU/mL penicillin). *A*. *fumigatus* WT (akuB^Ku80^ pyrG^+^) [34] and *Δugm1* mutant [35] strains were used. Other fungal and bacterial strains were obtained from the EMTC collection. Fungal cells, *Aspergillus fumigatus*, *Aspergillus flavus*, and *Candida albicans*, were propagated in Sabouraud medium at room temperature. *Enterococcus faecalis*, *Escherichia coli*, *Proteus mirabilis*, *Pseudomonas aeruginosa*, *Salmonella enterica*, and *Staphylococcus aureus* were grown in Luria-Bertani broth at 37°C, while *Bifidobacterium longum* and *Lactobacillus plantarum* were grown in Blaurock medium.

### Mice immunization and mAbs selection

For immunization, 12–14-week-old BALB/c mice (22–28 g) were subcutaneously administered with 15 μg BSA-conjugate **GM-1-BSA** (Fig 1B) in 300 μL phosphate buffer saline (PBS), pH 7.4, emulsified with an equal volume of complete Freund’s adjuvant (Sigma-Aldrich, USA). Each mouse was boosted twice at two-week and four-week intervals with equal amount of the immunogen mixed with incomplete Freund’s adjuvant (Sigma-Aldrich, USA) in the same ratio. The fusion titer of anti-galactomannan antibodies in mice sera was screened using indirect ELISA. Two weeks after the third hybridization, animals were finally administered with 20 μg BSA-conjugate **GM-1-BSA** (Fig 1B) in 300 μL PBS, pH 7.4. Three days after this procedure, mice with the highest fusion titer were sacrificed, spleens were obtained, and splenocytes were fused with SP2/0 myeloma cells using PEG 2000 (Roche, Switzerland) according to the manufacturer’s protocol. The level of mAbs in the supernatants was tested using indirect ELISA. Positive clones were additionally cloned two times by limiting dilution method.

### Oligosaccharide-specific indirect ELISA

For indirect ELISA, the wells of 96-well Pierce™ streptavidin-coated plate were coated with 50 ng/well of biotinylated pentasaccharide **GM-1-Biot** (Fig 1B) in 25 μM Tris-HCl pH 7.5 with 150 mM NaCl, 0.5% Tween-20, and 0.3% BSA and incubated at 4°C overnight. The supernatants from mouse experiments or mAbs in appropriate dilutions were added and incubated at 37°C for 1 h. After washing, anti-mouse IgG alkaline phosphatase-conjugated goat IgG (Sigma Aldrich, USA) was added and incubated at 37°C for 1 h followed by staining with 4-nitrophenyl phosphate. Absorbance was measured at 405 nm using iMark plate reader (Bio-Rad, USA). To exclude binding of mice sera and mAbs with BSA, the wells of 96-well microtiter plate were coated with control antigen, 3% BSA in PBS, pH 7.4, and after blocking, mice sera or mAbs were added and incubated at 37°C for 1 h. Then, indirect ELISA was conducted as described above.

### Purification and conjugation of mAbs

To produce mAbs, 2 × 10^6^ selected hybridoma cells were administered intraperitoneally into 20-week-old BALB/c mice. Selected mAbs were purified by ammonium sulfate precipitation from ascitic fluids and then purified using the protein A chromatography (GE Healthcare, USA). The purity and size of the purified IgG antibodies were examined by SDS-PAGE and western blot analyses. Purified mAbs were resolved by 12.5% SDS-PAGE under reducing conditions and transferred onto nitrocellulose membrane (Bio-Rad). After blocking by 5% dry skim milk, the membrane was incubated with anti-mouse IgG alkaline phosphatase-conjugated goat IgG (Sigma Aldrich, USA). Immune complexes were visualized by a mixture of nitro blue tetrazolium (NBT, Amresco) and 5-bromo-4-chloro-3-indolyl-phosphate (BCIP, Roche) for 20 min.

Selected mAbs were conjugated with horse-radish peroxidase (Amresco, USA) using optimized NaIO_4_ method as described previously [36].

### Affinity constant measurement

**Fig 2:**
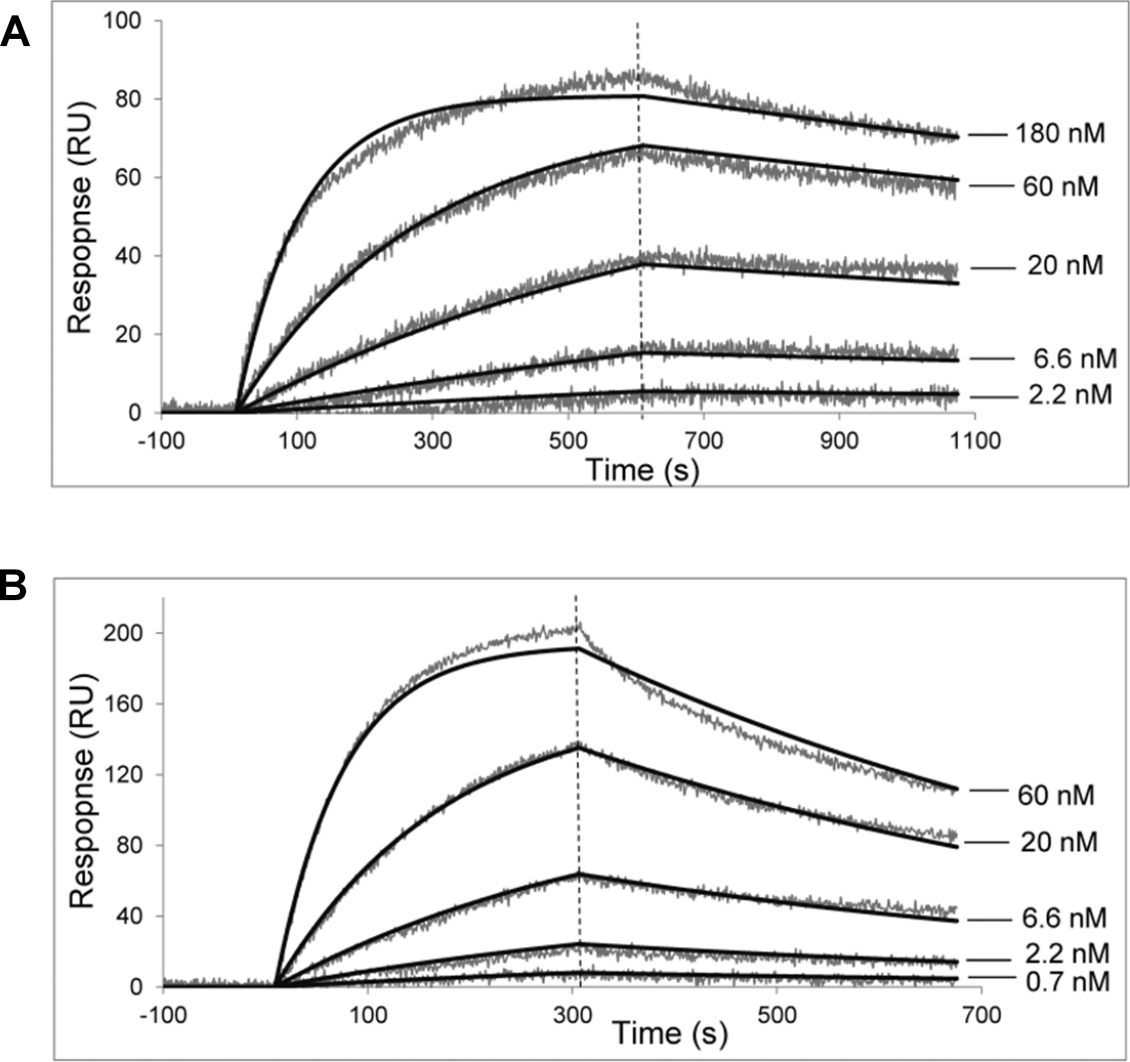
Binding of selected mAbs with biotinylated pentasaccharide GM-1. Serial three-fold dilutions of (A) mAb 7B8 starting from 180 nM and (B) mAb 8G4 starting from 60 nM were used as analytes. Fitted traces are depicted as smooth black lines. A global analysis of the interaction demonstrated a good quality fit and experimentally determined dissociation and association rate constants, k_d_ and k_a_, were (3.0 ± 0.1) × 10^−4^ s^−1^ and (5.6 ± 0.1) × 10^4^ M^−1^s^−1^ for 7B8 antibody, and (1.5 ± 0.1) × 10^−3^ s^−1^ and (2.3 ± 0.1) × 10^5^ M^−1^s^−1^ for 8G4 antibody, respectively. Equilibrium constants, calculated as K_D_ = k_d_/k_a_, were 5.3 × 10^−9^ M and 6.4 × 10^−9^ M, respectively.

The kinetics for mAbs binding to synthetic galactomannan oligosaccharides conjugated with biotin were determined by a surface plasmon resonance (SPR) using a ProteOn XPR36 system (Bio-Rad, USA). Vertical channels L1 and L2 of GLC sensor chip were coated with streptavidin at 150–180 response unit (RU) level. Tested biotinylated oligosaccharides were immobilized onto streptavidin-coated channel L1 at 10 RU level, while L2 was used as a reference channel. Serially diluted mAb was analyzed starting from the lowest concentration at a flow rate of 25 μL/min. Chip surface was regenerated with 100 mM citric acid. Global analysis of experimental data based on a single-site or a heterogeneous analyte model was performed using the ProteOn Manager v. 3.1.0 software. Affinity constants were calculated as K_D_ = k_d_/k_a_ (see Fig 2).

### Glycoarray

**Fig 3:**
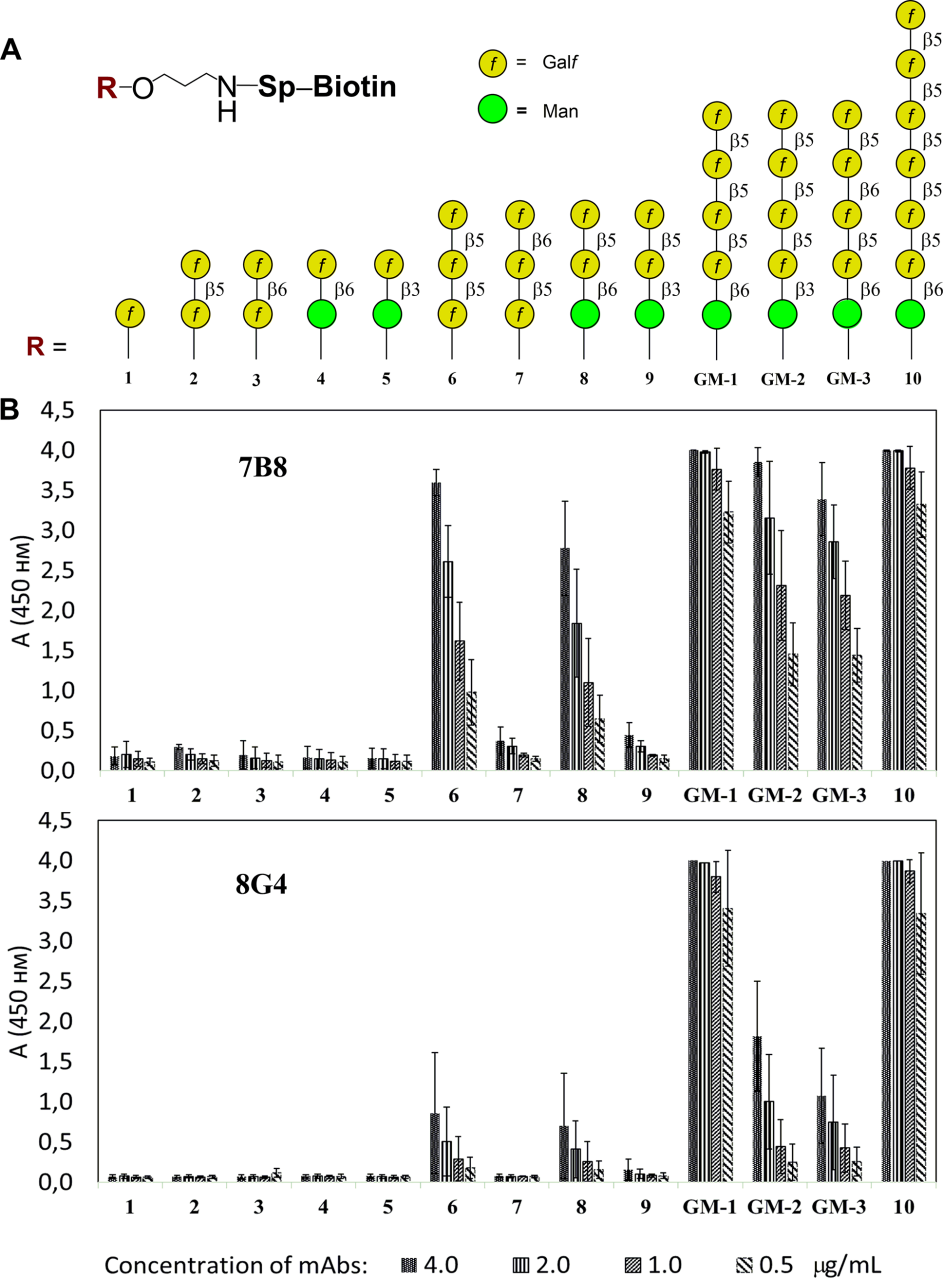
Investigation of oligosaccharide specificity of mAbs 7B8 and 8G4 using ELISA. (A) Composition of thematic glycoarray built using oligosaccharide ligands representing key structural elements of *A*. *fumigatus* galactomannan chain, and (B) assay for carbohydrate specificity of 7B8 and 8G4 mAbs.

The wells of 96-well Pierce™ streptavidin-coated plates were coated with appropriate biotin-tagged oligosaccharides **1–10**, **GM-1**, **GM-2**, **GM-3** (Fig 3) (100 μL of a 20 pmol/well solution in PBS containing 0.05% Tween-20 and 0.1% BSA) and then incubated for 2 h at 37°C. The plates were incubated with mAbs 7B8 and 8G4 serially diluted in PBS-BSA-Tween-20 buffer (two-fold, starting from 8 μg/mL concentration) for 1 h at 37°C. After washing, anti-mouse IgG rabbit IgG-horseradish peroxidase conjugate (Imtek, Russia) was added and incubated for 1 h at 37°C. After washing three times, color was developed using TMB mono-component substrate (100 μL) for 15 minutes and stopped with 50 μL of 1 M sulfuric acid. Absorbance was measured at 450 nm using MultiSkan GO plate reader (Thermo Fisher Scientific, USA). All measurements were done in triplicates.

### Microscopy

For fluorescent microscopy, *A*. *fumigatus* WT and *Δugm1* mutant strains were grown in Sabouraud broth at 30°C for 20 h. After fixation of the mycelium with 2.5% *p*-formaldehyde (PFA) overnight at 4°C, immunofluorescence procedure was performed as described previously [37]. Briefly, cells were washed with PBS, incubated with 2% glycerol/PBS for 5 min and 5% goat serum in PBS for 1 h. Then, cells were incubated with mAb (2 μg/mL in 5% goat serum/PBS) for 1 h at room temperature. After washing with goat serum/PBS, cells were incubated with a goat TRITC-conjugated Ab directed against anti-mouse IgG, (Sigma) diluted 1:200 in goat serum/PBS. After washing in PBS, cells were visualized under an inverted fluorescence microscope.

For confocal microscopy, fungal and bacterial cells were fixed with 2.5% PFA overnight at 4°C. Fixed cells were washed with PBS twice and incubated with 3% BSA in PBS for 1 h. Then, cells were washed and incubated with 5 μg/mL mAb diluted in PBS with 3% BSA for 1 h at 37°С. After washing, cells were stained with Alexa Fluor-488-conjugated chicken anti-mouse IgG (H+L) antibodies (Life Technologies) diluted 1:500 in PBS with 3% BSA. Samples were mounted using Prolong Diamond Antifade. Images were obtained using the Carl Zeiss LSM 710 laser scanning microscope (Carl Zeiss, Germany). Observations were done using oil 63× objectives and scans were taken at 488 nm in green and differential interference contrast (DIC) channels. ZEN black edition software (Carl Zeiss, Germany) was used in the confocal microscope to visualize images. Alexa-Fluor 488 images and DIC images were obtained by excitation at 488 nm, with emission collected from 510–540 nm.

### Sandwich anti-galactomannan ELISA

The wells of 96-well microtiter plates were coated with a selected 7B8 or 8G4 mAb in carbonate-bicarbonate buffer, pH 9.2 and then incubated at 37°C for 1 h. After blocking, serially diluted (two-fold, starting from 1:100 dilution) microbial supernatants in PBS-BSA-Tween-20 buffer were added, and the plates were incubated for 1 h at 37°C. After washing three times, indirect ELISA was performed with horseradish peroxidase-conjugated mAb (100 μL) and developed using TMB mono-component substrate (100 μL) for 15 minutes and stopped with 50 μL of 1 M sulfuric acid. Absorbance was measured at 450 nm using iMark plate reader (BioRad, USA). All microbial supernatants were tested three times and in triplicates.

## Results and discussion

### Production of anti-galactomannan mAbs

To generate mice mAbs specifically recognizing *A*. *fumigatus* galactomannan, the immunogen **GM-1-BSA** bearing pentasaccharide ligands comprising four (1→5)-linked galactofuranoside units attached via (1→6)-linkage to mannose residue was selected as a specific target fragment in the galactomannan structure (Fig 1A). The selected pentasaccharide represents characteristic fragment in the structure of *A*. *fumigatus* galactomannan [6,8]; it was shown that this pentasaccharide sequence can be used as a mimetic for *Aspergillus* galactomannan in immunobiological studies [38,39]. Using synthetic pentasaccharide **GM-1**, immunogen **GM-1-BSA** was prepared by conjugation of corresponding aminopropyl glycoside with BSA using the squarate protocol (Fig 1B). The biotin-tagged pentasaccharide **GM-1-Biot** required for selection of mAbs was prepared by treating amine with activated ester (Fig 1B).

To develop mAbs against galactomannan from the cell wall of *A*. *fumigatus*, BALB/c mice were immunized with BSA-conjugate **GM-1-BSA** four times. Four, 8, and 12 weeks after the first immunization, mice sera were screened by indirect ELISA to assess the level of anti- galactomannan IgG antibodies; biotinylated pentasaccharide **GM-1-Biot** was used as an antigen to exclude selection of anti-BSA mAbs. Final fusion titer of anti-**GM-1** antibodies in mice sera was 1:10 000 (data not shown). The binding of individual hybrid clones (n = 480) with biotinylated pentasaccharide **GM-1-Biot** was tested by indirect ELISA, and nine hybridomas were selected.

To determine IgG class of selected mAbs, a fragment of the gene encoding constant domain CH1 was amplified using the primers 5′-CTTCCGGAATTCSARGTNMAGCTGSAGSAGTC-3′ [40] and 5′-GGGAAGTAGCCTTTGACAAGGC-3′ and sequenced. Among nine selected mAbs, two belonged to the IgG3 class, while others belonged to the IgG1 class. Light chains of all the selected mAbs belonged to the kappa family.

All the selected mAbs were produced in ascitic fluids, purified using affinity chromatography, and visualized by PAAG and western blotting S1 Fig. The kinetic parameters and affinity constants for the interaction between the selected mAbs and target pentasaccharide were determined with biotinylated conjugate **GM-1-Biot** in a label-free biosensor assay using a ProteOn XPR36 system. A global analysis of interaction between the mAbs 7B8 and 8G4 and the antigen demonstrated a good quality fit and affinity constants were calculated as K_D_ = (5.3 ± 0.2) × 10^−9^ M for mAb 7B8 and K_D_ = (6.4 ± 0.2) × 10^−9^ M for mAb 8G4 (Fig 2). Affinity constants of other selected mAbs were > 10^−6^ М, which were insufficient for the development of EIA with good sensitivity for the detection of aspergillosis; therefore, further experiments were performed with mAbs 7B8 and 8G4.

### Epitope specificity of selected mAbs

The carbohydrate specificity of mAbs 7B8 and 8G4 was investigated using a library of 13 synthetic oligosaccharides representing distinct structural fragments of *A*. *fumigatus* galactomanan (Fig 3A). These ligands with varied size and types of inter-unit linkages represent the biotinylated conjugates, which were immobilized on the surface of the streptavidin-coated plate. mAbs were applied on glycoarrays as a series of two-fold dilutions. The highest affinity was demonstrated for the target pentasaccharide **GM-1** used in the structure of the immunogen, and in the case of elongated analog **10**, containing pentasaccharide sequence (Fig 3). Mono- and disaccharides were not recognized by both 7B8 and 8G4.

The discernible difference between mAbs 7B8 and 8G4 was revealed using the analogs of the target pentasaccharide **GM-1**. The shorter ligands, trisaccharides **6** and **8**, and ligands with changed type of linkages, **GM-2** and **GM-3**, were recognized moderately by mAb 7B8; however, its interaction with mAb 8G4 was significantly lower (Fig 3). Notably, trisaccharides **7** and **9**, structures of which were not presented in the target pentasaccharide, were not bound by both 7B8 and 8G4 (Fig 3).

These results were additionally confirmed by SPR analysis for determining the binding of mAbs 7B8 and 8G4 with pentasaccharides **GM-2** and **GM-3**, which differed from the target pentasaccharide **GM-1** with only one glycoside bond (Fig 1C). The affinities with which mAb 7B8 bound to pentasaccharides **GM-2** and **GM-3** were K_D_ = (5.9 ± 0.2) × 10^7^ M^-1^ and K_D_ = (3.3 ± 0.1) × 10^7^ M^-1^, respectively, and were lower than that for the initial pentasaccharide **GM-1** (see Fig 2). Notably, binding of mAb 8G4 to pentasaccharides **GM-2** and **GM-3** were two orders lower than that with the initial pentasaccharide **GM-1**, K_D_ = (6.7 ± 0.2) × 10^6^ M^-1^ and K_D_ = (2.0 ± 0.1) × 10^6^ M^-1^, respectively.

### Specific binding of mAbs with fungal and bacterial cells in culture

**Fig 4:**
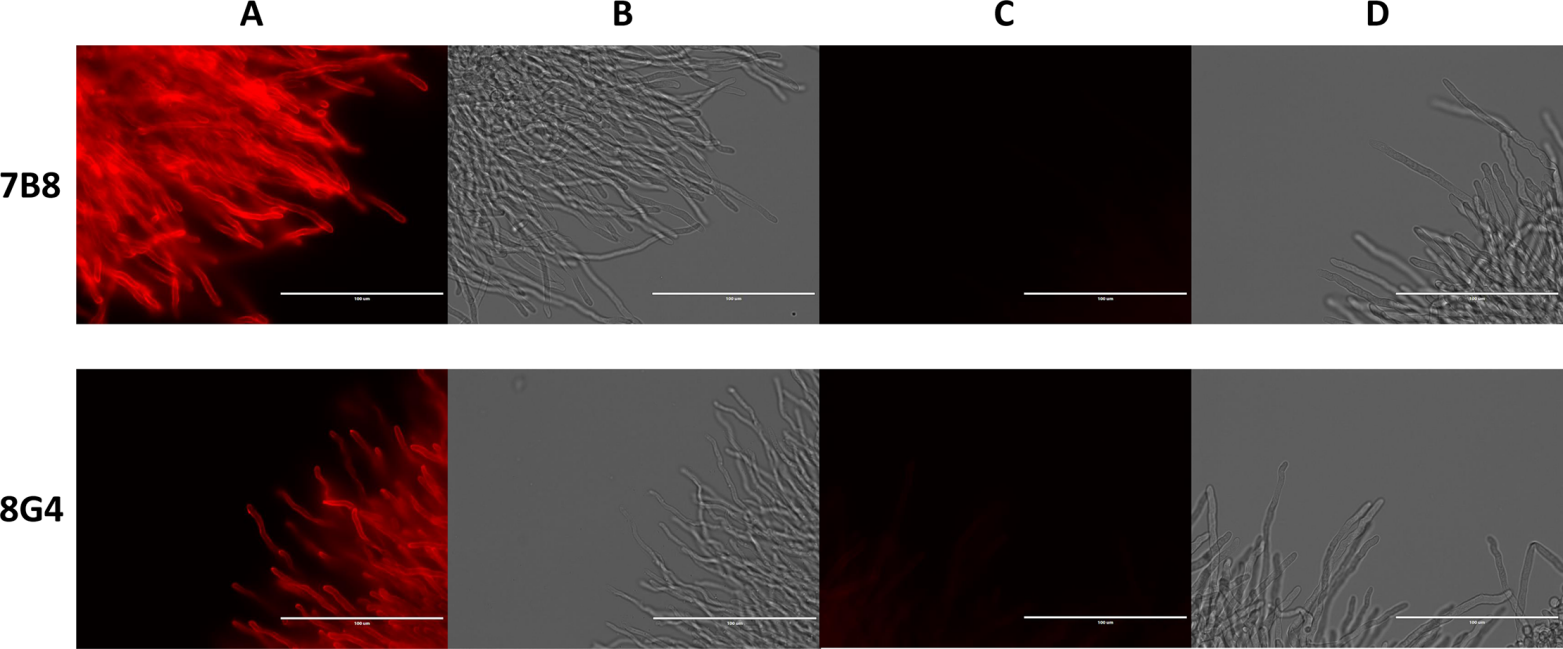
Immunofluorescence labeling of the *Aspergillus fumigatus* WT strain and *ΔUgm1* mutant with mAbs 7B8 and 8G4. (A, B) Wild-type parental strain of *A*. *fumigatus* in fluorescence microscopy and light microscopy studies, respectively. (C, D) *Ugm1* mutant of *A*. *fumigatus* without galactomannan in fluorescence microscopy and light microscopy studies, respectively. Binding of mAbs with Aspergillus cells was detected with goat TRITC-conjugated anti-mouse IgG antibody. Scale bar = 100 μm.

To demonstrate the ability of mAbs 7B8 and 8G4 to specifically recognize natural galactomannan, immunofluorescence experiments were performed using parental *A*. *fumigatus* WT and *Δugm1* mutant strains (Fig 4). Notably, previously it has been demonstrated that mycelial cell wall of *A*. *fumigatus Δugm1* mutant does not contain galactofuranose [35]. In this study, both 7B8 and 8G4 mAbs labeled *A*. *fumigatus* parental strain, while no fluorescence signal was observed for the *Δugm1* mutant (Fig 4). The lack of fluorescence signal demonstrated the involvement of galactofuranoside unit in epitopes recognized by mAbs 7B8 and 8G4.

**Fig 5:**
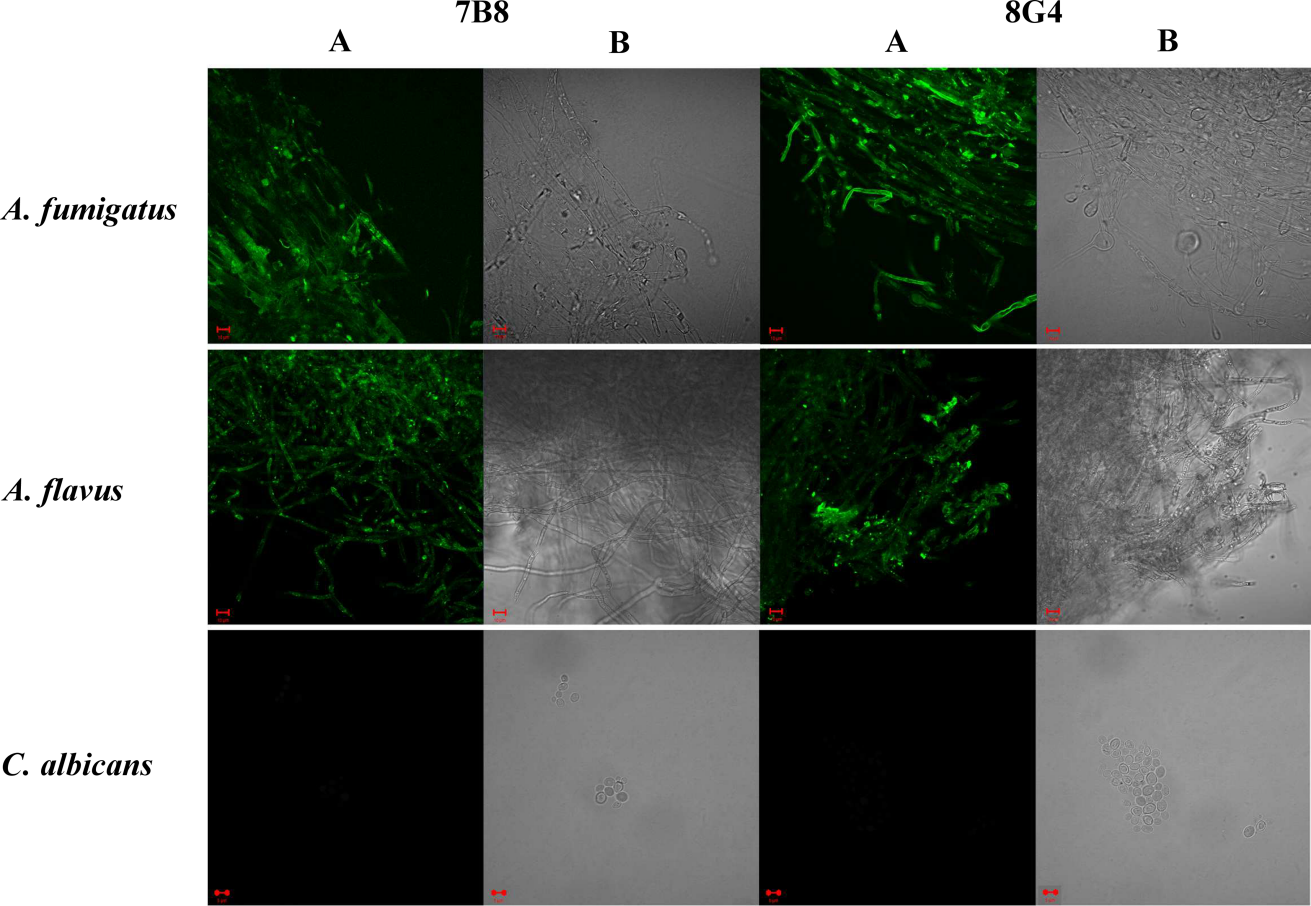
Specific binding of mAbs 7B8 and 8G4 with *A*. *fumigatus*, *A*. *flavus*, and *C*. *albicans*. Cells were grown in Sabouraud broth, fixed, and incubated with mAbs 7B8 and 8G4. Binding of mAbs with fungal cells was detected with Alexa Fluor 488-conjugated anti-mouse IgG antibody staining in (A) confocal microscopy, and (B) DIC microscopy. Scale bar = 10 μm.

To evaluate specificity of mAbs 7B8 and 8G4, their binding with fungi *A*. *fumigatus*, *A*. *flavus*, and *C*. *albicans*, as well as gram-positive and gram-negative bacterial cells, including *B*. *longum*, *E*. *faecalis*, *E*. *coli*, *L*. *plantarum*, *P*. *mirabilis*, *P*. *aeruginosa*, *S*. *enterica*, and *S*. *aureus*, was examined by confocal microscopy. Previously, it has been demonstrated that some fungal and bacterial species express polysaccharides that have structural elements similar to those from *A*. *fumigatus* galactomannan, while some of them do not express such polysaccharides [34,35]. In case of *A*. *flavus*, structure of carbohydrate antigens has not been studied in all details, however the previous immunological studies [19] suggest the presence of structures related to A. fumigatus galactomannan. The obtained images demonstrated that both 7B8 and 8G4 labeled *A*. *flavus* along with *A*. *fumigatus* (Fig 5). However, *C*. *albicans* (Fig 5) and all the tested bacteria (S2 Fig) were not detected by mAbs 7B8 and 8G4. Importantly, exposure time was the same in all experiments.

**Fig 6:**
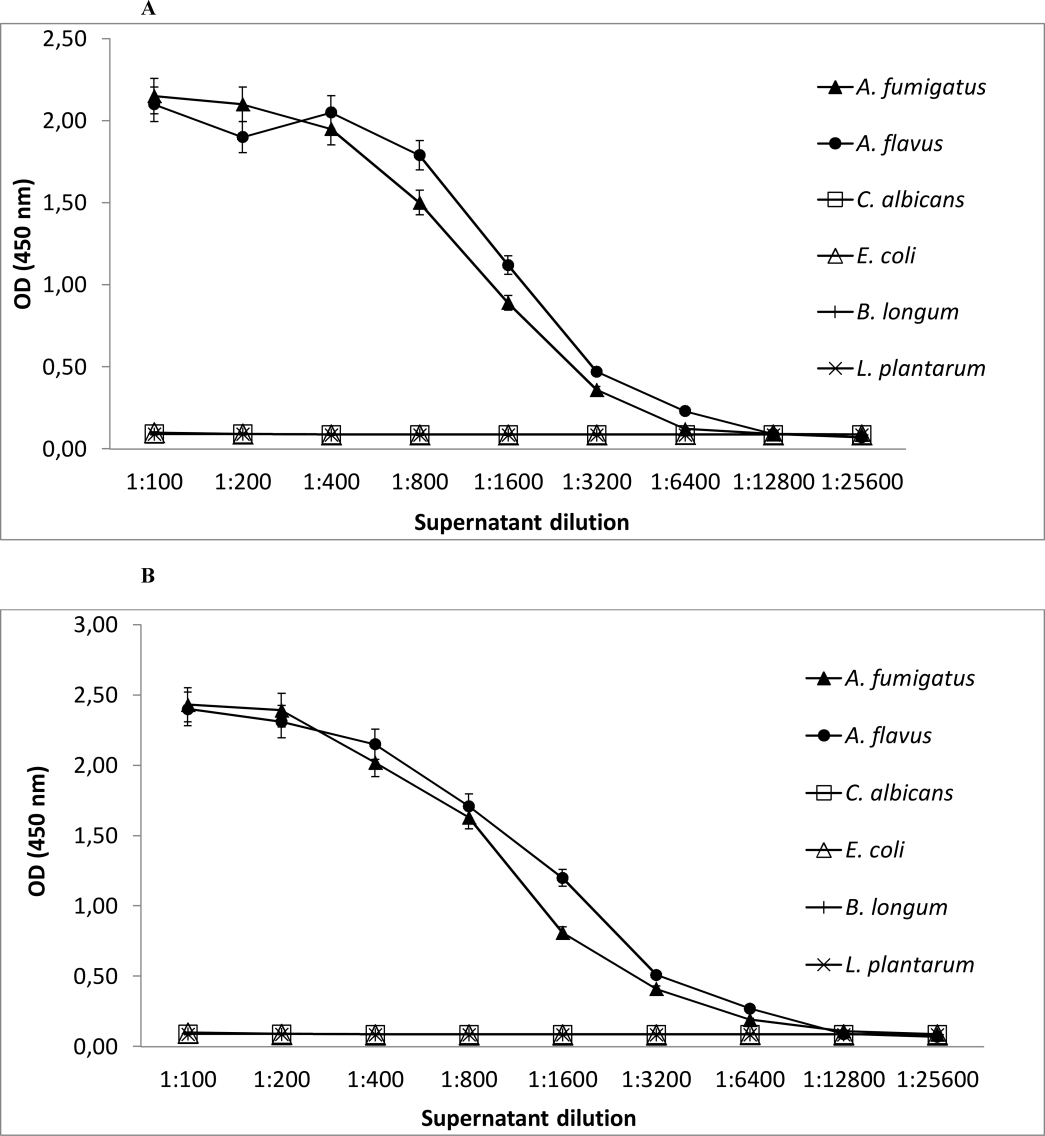
Binding of fungal and bacterial cultures with mAbs 7B8 and 8G4. (A) Sandwich enzyme-linked immunosorbent assay (ELISA) with 7B8 mAb: the wells of microtiter plates were coated with 7B8 mAb and incubated with serial dilutions of microbial supernatants; ELISA was performed with horseradish peroxidase-conjugated 7B8 mAb. (B) Sandwich ELISA with 8G4 mAb: the wells of microtiter plates were coated with 8G4 mAb and incubated with serial dilutions of microbial supernatants; ELISA was performed with horseradish peroxidase-conjugated 8G4 mAb.

Specific binding of mAbs 7B8 and 8G4 with *A*. *fumigatus* and *A*. *flavus* and the lack of their binding with *C*. *albicans*, *B*. *longum*, *E*. *faecalis*, *E*. *coli*, *L*. *plantarum*, *P*. *mirabilis*, *P*. *aeruginosa*, *S*. *enterica*, and *S*. *aureus* were confirmed using sandwich ELISA (Fig 6). In accordance with the confocal microscopy data, supernatants of *A*. *fumigatus* and *A*. *flavus* added to mAb 7B8 were effectively bound by the same mAb (Fig 6A) or mAb 8G4 (data not shown), while supernatants of *C*. *albicans*, *B*. *longum*, and other tested bacteria were not recognized by sandwich ELISA (data for *E*. *faecalis*, *P*. *mirabilis*, *P*. *aeruginosa*, *S*. *enterica*, and *S*. *aureus* are not shown). The same result was demonstrated for sandwich ELISA with mAb 8G4 (Fig 6B).

Importantly, both mAb 7B8 and 8G4 could not detect *B*. *longum* cells either by confocal microscopy, or by sandwich ELISA. However, *Bifidobacterium* spp., including *B*. *longum* strains, have previously demonstrated substantial cross-reactivity with sandwich ELISA based on EB-A2 mAb [25]. *Bifidobacterium* species comprise a considerable part of the normal gastrointestinal microflora of adults and reach 90% of the total fecal microflora of infants [41,42]. Translocation of membrane-associated galactofuranoses because of the immaturity of the intestinal mucosa in neonates can explain some false-positive results [24]. The lack of binding of the mAbs 7B8 and 8G4 with *Bifidobacterium* species indicates high specificities of the studied mAbs, which could be further tested head-to-head with EB-A2 as a promising components for the development of a new specific enzyme-linked assay for detection of *A*. *fumigatus*, required for medical and environmental uses.

## Conclusions

Mouse mAbs 7B8 and 8G4, which efficiently recognize galactomannan of *A*. *fumigatus*, were obtained by immunization of mice with BSA-conjugate of synthetic pentasaccharide β-D-Gal*f*-(1→5)-[β-D-Gal*f*-(1→5)]_3_-α-D-Man*p* (**GM-1**) and hybridoma technology. The carbohydrate specificity of obtained mAbs was assessed by SPR and thematic glycoarray built using a series of synthetic oligosaccharide ligands structurally related to the characteristic galactomannan fragments. Further studies on staining of fungal and bacterial cells by mAbs confirmed good selectivity of developed mAbs suitable for detecting *A*. *fumigatus* galactomannan and made possible their use in immune diagnostics. Results showed that because of recent progress [38,43] in the synthesis of oligosaccharides related to fungal cell wall carbohydrate antigens, the synthetic oligosaccharide derivatives of distinct structure can be efficiently used for the development of mAbs as an alternative to natural polysaccharides, which are characterized by structural diversity.

## Supporting information

S1 Fig Electrophoretic and western blot analyses of monoclonal antibodies 7B8 and 8G4. (A) Coomassie blue stained 12% SDS–PAAG electrophoretic analysis of purified mAb 7B8 and mAb 8G4 in reducing conditions. (B) Western blot analysis of mAb 7B8 and mAb 8G4 fractionated by 12.5% SDS-PAAG electrophoresis in reducing conditions and developed with alkaline phosphatase conjugated anti-mouse IgG (whole molecule) goat antibody (Sigma-Aldrich, USA). Protein molecular marker masses, in kilodaltons, are shown at the left side of the gel.
(TIF)

S2 Fig Luck of binding of monoclonal antibodies 7B8 and 8G4 to selected bacterial cells. Fixed cells were incubated with mAbs 7B8 and 8G4. (A) Bacterial DNA was stained with DAPI. (B) Binding of mAbs with bacterial cells was identified with Alexa Fluor 488 conjugated anti-mouse IgG antibodies staining in confocal microscopy. (B) Scale bar 10 μm.
(TIF)
