# Supplementary figures and images for: Novel mouse monoclonal antibodies specifically recognize Aspergillus fumigatus galactomannan
Source: PLoS One. 2018 Mar 8;13(3):e0193938. doi: 10.1371/journal.pone.0193938 (PMC5843280; doi:10.1371/journal.pone.0193938)

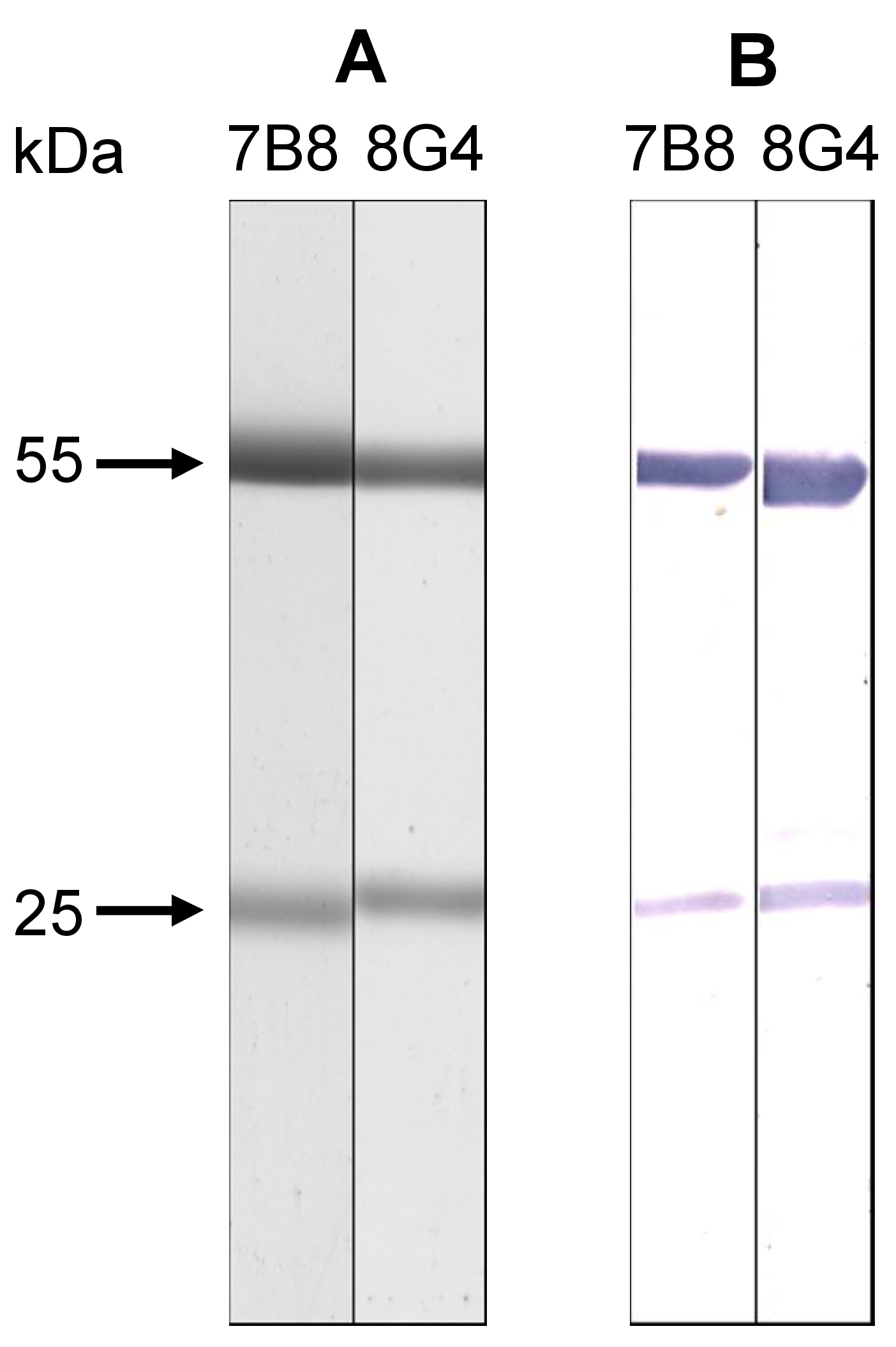

Supplement: S1 Fig — (A) Coomassie blue stained 12% SDS–PAAG electrophoretic analysis of purified mAb 7B8 and mAb 8G4 in reducing conditions. (B) Western blot analysis of mAb 7B8 and mAb 8G4 fractionated by 12.5% SDS-PAAG electrophoresis in reducing conditions and developed with alkaline phosphatase conjugated anti-mouse IgG (whole molecule) goat antibody (Sigma-Aldrich, USA). Protein molecular marker masses, in kilodaltons, are shown at the left side of the gel. (TIF) [file pone.0193938.s001.tif]

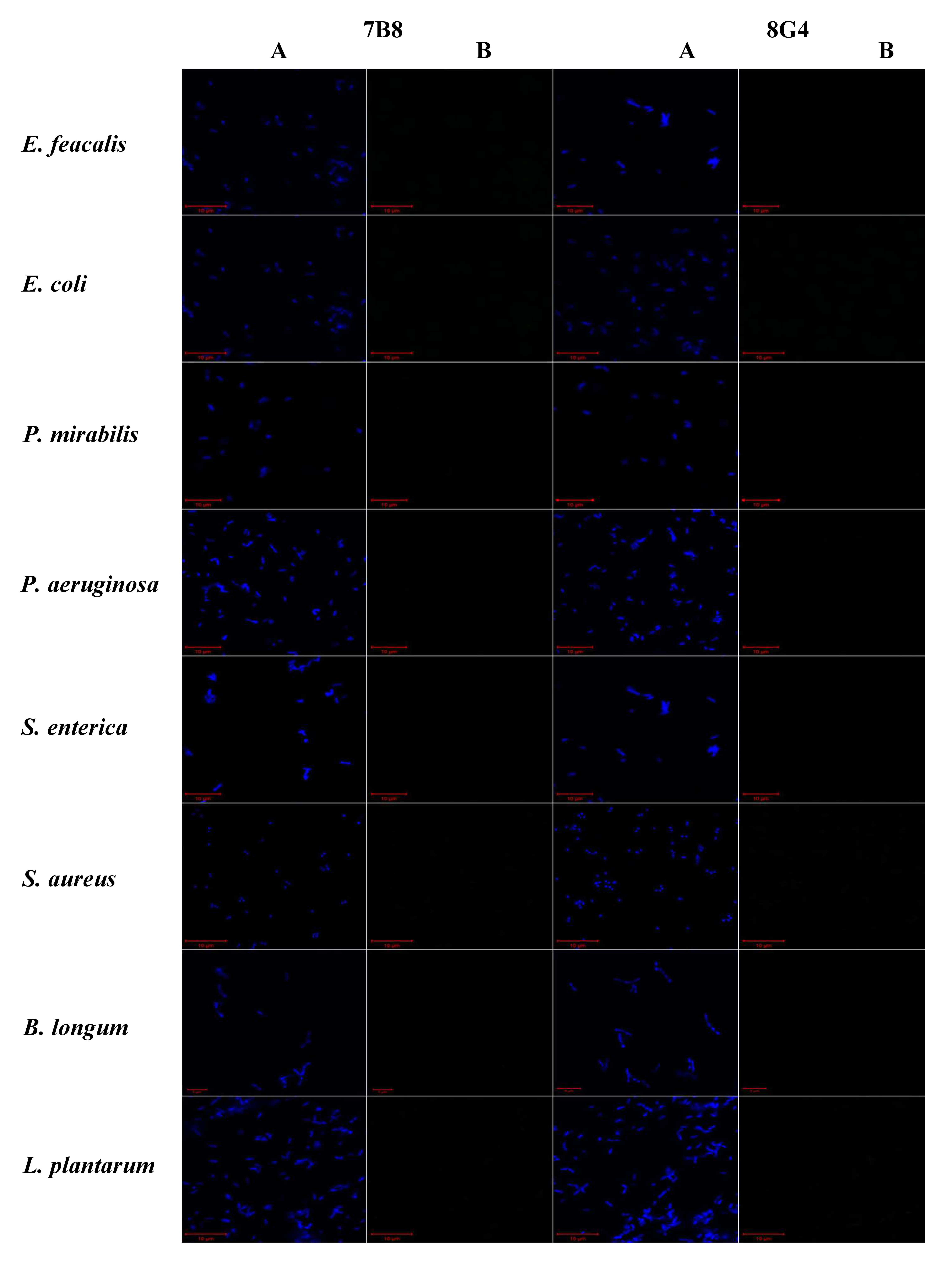

Supplement: S2 Fig — Fixed cells were incubated with mAbs 7B8 and 8G4. (A) Bacterial DNA was stained with DAPI. (B) Binding of mAbs with bacterial cells was identified with Alexa Fluor 488 conjugated anti-mouse IgG antibodies staining in confocal microscopy. (B) Scale bar 10 μm. (TIF) [file pone.0193938.s002.tif]
